# The Hygiene of Old Age

**Published:** 1886-06-20

**Authors:** H. C. Wood


### THE HYGIENE OF OLD AGE.

By H. C. Wood, M. D.

Within a few weeks the city of Philadelphia has been called upon to mourn the loss of the man who, although very far from intellectually the greatest within her borders, as a citizen was preeminently chief. Dying at the age of eighty or eighty-one years, he is universally spoken of as being gathered like a ripened sheaf; yet, within a week of his burial, he was full of mental and physical vigor, and his death at the time was as unnecessary and avoidable as though he had only reached threescore years. A very notable percentage of the deaths of persons who have been successful in life, and have attained beyond the seventieth year, could be, by proper care, long postponed. Failure in life in a large proportion of cases saps vitality, and the man who carries the load of self-knowledge of such failure lives under a persistent strain, whose effects, though usually not recognized, are none the less irresistible. In order to protract an advanced life it is well to understand not only the dangers that beset such life, but the reason why old age has been attained.

The humorist is greatest when underlying his rollicking is the lesson of a great truth; but perhaps few readers, when they enjoyed the broad fun of the "One-Horse Shay," as portrayed by our inimitable Holmes, have recognized the fact that the man who reaches old age dose so largely because he has been constructed npon the principles of the famous vehicle " that ran for a hundred years and a day." Barring accidental deaths from railroad collisions, typhoid fevers, lightning-strokes, and other more or less preventable causes, the man who is so built that he is equally strong in all his parts, lives out his appointed days.

Excessive strength in one part is a veritable source of danger. The athlete perishes because his over-developed muscular system perpetually strains and finally wears out a heart or lung that was originally constructed for a muscular apparatus of half the power of that which he has artificially built up. The larger proportion of mankind die early on account of some local weakness. It ought to be generally recognized that human age is not to be counted by years, and that in some constitutions the general tissues are older at fifty than they are in other individuals at one hundred. Many of the cases of so-called neurasthenia, or nervous exhaustion, of men and women suddenly or gradually breaking down at forty or fifty, ostensibly from overwork, are really cases of premature old age, and are to be nursed and treated precisely as other individuals would be who had reached to fourscore years. Moreover, a large proportion of early deaths are the result of some vital organ being originally endowed with a longevity less than that of the rest of the organism. The reason that consumption is so often utterly irremediable is to be found in the fact that in not a few cases the lung has reached its alloted term of days, and must die because its vitality is exhausted. If an eye, or other not vital part, fails from lack of vital power, the man exists; but if a lung dies, he perishes.

The result of these lucubrations is to lead us to this point, namely, that the individual who enjoys fair health at seventy-five years of age has probably been built upon the principle of the "One-Horse Shay," and that he should be treated as a wise man would treat such a venerable instrument of progression. He would certainly keep it off Philadelphia cobble-stones, and allow it only to be bowled along some smooth turnpike, and especially would he avoid all jolts and jars which would throw an unexpected strain upon one part. The principle involved in such case is that which is most vital in the treatment of the old—protection, and especially protection from strain of any one vital part. An old man exposes himself to inclement weather, and especially to a high wind, which suddenly drives the blood from the surface upon the internal organs, and at the same time by its very force checks the enfeebled movements of respiration, which aid in forcing the blood out from those organs. As a result, the man perishes at once, because he has thrown too great a strain upon a weak heart, or, if able to momentarily resist the strain, dies in a few days of pneumonia, due to the congestion of the lung. I have known the sudden shock of good news to strike the old man down, as fatally as the pole-axe fells the bullock, by causing the blood to rush with renewed force through the brain, and tear its way through the weakened walls of the blood-vessels. Again, the violent emotion of a sudden bad news may overwhelm a heart which, with care, would have sufficed for its duties for many years. The young athlete in the boat race pulls at his oar until he drops from heart-strain, and, if the heart-strain has not been too severe, recovers himself in a few weeks, because the vital elasticity of the heart-tissues is in highest vigor. But the enfeebled and brittle heart-muscle of the old man, strained in some hurried effort to catch a railroad train, or in some equally unreasonable procedure, has no power of recovery, and rests itself only in death. What is true in regard to the healthy ordinary conditions of the old man is more abundantly true in regard to the diseases of the old. Medicines that perturbate—measures that bring relief through violent local actions cannot be borne, and are not to be employed. At the same time, when possible, it is most essential to arrest at once any incipient disorder in the aged. I knew an old doctor, renowned in all lands, who lived ten years beyond the period attained before any one of his name, largely because, knowing himself thoroughly, every few weeks he arrested in its inception an attack, which, in a few hours, might have gathered fatal force.

I feel some hesitation in attempting to point out in detail the application of the principles which have just been enunciated, lest this paper may fall into the hands of aged persons, and be substituted for a careful consideration of their individual cases by some skilful medical practitioner. Every person, when he advances in years, should go over his whole methods of life and personal habits with some wise counsellor, and should adapt his mode of life to the peculiarities of his individual case. With this warning, it is probably safe to briefly point out some of the more important details in the regulation of the life of old people. The first question is in regard to food. The teeth in old age are, of course, lost, and they should, unless under exceptional circumstances, be replaced by artificial teeth, for the thorough chewing of food is even mere necessary in the old than in the young, because in the old the digestive powers are apt to fail. With the best artificial teeth mastication is apt to be imperfectly performed ; hence the food of the aged should be soft and readily comminuted, and especially should it be of easy digestion. Very few old people need stimulating diet; very many are injured by an excess of nitrogeneous food. The kidneys, like all other organs, are feeble, and if meats and other rich foods are used in excess, they greatly increase the strain upon these organs. Milk and milk products, or preparations of breadstuffs cooked with milk, should form a very large proportion of the food of the ordinary aged individual; but individual peculiarities differ so much that personal medical counsel should in all cases be taken, so that the diet may be regulated to the needs of the individual case. Very many old people are hurt by the use of food in excessive quantity; but little exercise can be taken, all growth has ceased, and the bodily furnaces which make heat are able to destroy but very little of food fuel. Some little time since I had occasion to lecture on this subject at the Philadelphia Hospital and an assertion that I then made that most old people are more comfortable, enjoy better health, and probably live longer for the use of wine, has met with very severe disapprobation at the hands of some of the profession, whose strong sympathy with the temperance movement dominates their judgment. No valid reasons have, however, so far as my judgment goes, been brought forward to lead me to change my opinion. In the overfed American people the habitual use of wine during youthful or middle age and vigorous health is, we think, an injury rather than a good ; but when the powers of life are failing, when digestion is weak, and the multitudinous small ills of feebleness perplex and annoy, one or two glasses of generous wine at dinner aid digestion, quiet for the time being much nervous irritation, and in no way do harm. The sum total of ruin wrought by alcohol in the world is appalling, but it is not lessened by our shutting our eyes to the good that wine properly used may achieve. When in the aged there is a distinct failure of vital power, and especially of digestive power, the call for the habitual use of alcoholic liquors is, in my opinion, imperative. The danger of the formation of any evil habits when a man has crossed the line of seventy is so slight, that the most conscientious physician need not hesitate recommending the daily use of alcoholic beverages to his patient.

It is, perhaps, not universally recognized, that in numerous cases of various character death finally is due, in greater or less measure, to cold and to an absolute failure on the part of the body to keep itself warm. In the old the heat-making functions are exceedingly low, and hence it is that few old people are comfortable in a room whose temperature is less than 80°. It is especially important, therefore, that an abundance of clothes be worn by old people; but the very weight of clothes oppresses, so that it is important that lightness of material should be combined with warmth. There is no ordinary garment which compares in heat-preserving powers with the buckskin jacket, and, in our climate, every man who passes the seventieth year should furnish himself with such covering. At first the jacket should be only worn when going out of doors; but in very advanced age it should form a part of the habitual underwear. The jacket should be high up in the neck and long in the sleeves, and should be of such a length as to thoroughly cover the abdomen. If worn as an under-jacket, it should be perforated so as to allow the escape of the vaporous emanations from the body. Whenever there is any tendency to abdominal weakness, in addition to the jacket and the ordinary warm underclothes, an abdominal flannel bandage should be worn. It ought not to be forgotten that the mass of blood of the human body is in the abdominal organs, and that this is especially so when the circulation is sluggish. It is affirmed by authority, that after death all the blood of the body can be put in the relaxed abdominal vessels; hence the importance of maintaining the abdominal warmth, and hence also the good effect in feeble people with pendulous bellies of the bandage, which helps sustain the relaxed vessels, and thereby maintain the general circulation. The mechanical effects of tight bandages are well understood by the profession in the treatment of ascites. It is well known that the sudden removal of the fluid by tapping over the abdominal cavity take away so much pressure from the abdomieal vessels as to cause them to relax, and draw the blood away from the heart and lungs and brain in sufficient quantity to produce fainting. It is to prevent this that the patient about to be tapped is bound up, and the bandage continually tightened as the water flows off. The importance of the habitual abdominal bandage is, perhaps, no less although not as universally recognized.—*Therapeutic Gazette.*

